# Prevalence of tuberculosis and associated factors among presumptive TB refugees residing in refugee camps in Ethiopia

**DOI:** 10.1186/s12879-023-08469-5

**Published:** 2023-07-28

**Authors:** Abyot Meaza, Bazezew Yenew, Miskir Amare, Ayinalem Alemu, Michael Hailu, Dinka Fikadu Gamtesa, Mirgissa Kaba, Girmay Medhin, Gobena Ameni, Balako Gumi

**Affiliations:** 1 Aklilu Lemma Institute of Pathobiology (ALIPB), Addis Ababa University (AAU), P.O. Box 1176, Addis Ababa, Ethiopia; 2 Ethiopian Public Health Institute (EPHI), Swaziland Street, PO Box 1242, Addis Ababa, Ethiopia; 3 School of Public Health, Addis Ababa University, Addis Ababa, Ethiopia; 4 Department of Veterinary Medicine, College of Agriculture and Veterinary Medicine, United Arab Emirates University, PO Box 15551, Al Ain, UAE

**Keywords:** Prevalence, Tuberculosis, Presumptive, Refugees, Refugee camps in Ethiopia

## Abstract

**Background:**

Tuberculosis (TB) causes significant morbidity and mortality in refugee populations. Although Ethiopia is the third largest refugee-hosting country in Africa, there is limited published data on the prevalence and associated factors of TB in refugees. The objective of this study was to estimate the prevalence of bacteriologically confirmed pulmonary TB (PTB) and explore associated factors in presumptive TB refugees residing in refugee camps in Ethiopia.

**Methods:**

A facility-based cross-sectional study was conducted between February and August 2021 in refugee camps in Ethiopia. Data were collected consecutively from 610 presumptive TB refugees who attended for TB diagnosis in selected refugee camp clinics in Ethiopia. A pre-tested questionnaire was used to collect data, and sputum samples were collected from eligible study participants. The Xpert *Mycobacterium tuberculosis* (MTB)/Rifampicin (RIF) assay was performed on direct spot sputum samples, whereas morning sputum samples were processed and inoculated for bacteriological culture using Mycobacterium Growth Indicator Tube (MGIT) and Lowsteen Jensen (LJ) methods. The statistical software package (STATA version 14) was used for statistical analysis. A logistic regression model was used for the evaluation of the association between bacteriologically confirmed TB cases and the associated factors. Descriptive statistics were used for the expression of the results, and statistical significance was assumed at p < 0.05.

**Results:**

Out of 610 study participants, more than half were female (54.9%), and the mean age was 37.9 years (SD, 16.64). The prevalence of bacteriologically confirmed PTB cases among refugees residing in refugee camps in Ethiopia was 13.3% (95% CI, 10.7–16.2%) using the Xpert MTB/RIF assay and/or culture. MTB was detected in 12.8% (95% CI, 10.2–15.7%) of the individuals using the Xpert MTB/RIF assay, while culture positivity was observed in 11.6% (95% CI, 9.2–14.5%). The multivariable logistic regression model showed South Sudan origins (adjusted odds ratio, AOR = 7.74; 95% CI, 3.05–19.64), age group, 19–38 years old (AOR = 5.66; 95% CI, 1.86–17.28), and male sex (AOR = 2.69; 95% CI, 1.58–4.56) were significantly associated with the bacteriologically confirmed TB among refugees residing in refugee camps in Ethiopia.

**Conclusion:**

The prevalence of bacteriologically confirmed PTB among presumptive TB refugees residing in refugee camps in Ethiopia was high. The national TB program should strengthen TB prevention and control activities in the refugee camps of Ethiopia. Moreover, an active TB survey program should be implemented in refugee camps in Ethiopia.

## Introduction

Tuberculosis (TB) is a major public health problem throughout the world, infecting an estimated quarter of the world’s population and putting them at risk of developing active disease during their lifetime [1]. TB is a communicable disease that causes ill health and it is one of the leading causes of death worldwide [1]. The recent World Health Organization (WHO) report indicated 10.6 million new cases and 1.6 million deaths occurred in 2021 globally [1], while 2.5 million new cases and more than a half million deaths were reported from the WHO African region [1]. There are existing guidelines and strategies for TB diagnosis [2], treatment [3], prevention, and control [4, 5] to combat the TB epidemic. Ethiopia remains among the global high TB burden countries, with an annual TB incidence of 119 cases per 100,000 population in 2021 [6]. Detection of TB cases and linkage to TB treatment are the key intervention areas of the TB prevention and control program in Ethiopia [6].

TB is an important cause of morbidity and mortality among refugees [7]. Conflict is the most common cause of displacement of a large population, which often results in relocation to temporary settlements (e.g., camps) with a significant risk of exposing people to becoming refugees [7]. Refugees refer to people who are outside their country and cannot return owing to a well-founded fear of persecution because of their race, religion, nationality, political opinion, or membership in a particular social group [8]. Factors including malnutrition and overcrowding in camp settings increase the vulnerability of displaced populations [7]. TB is one of the infectious diseases that can affect refugees due to poor living conditions and overcrowding [9]. Consequently, the incidence and prevalence of TB among refugees and migrant populations are higher than among non-refugee populations [10]. In a recent systematic review, the incidence and prevalence of TB ranged from 19 to 754 cases per 100,000 population and 18.7 to 535 cases per 100,000 population, respectively [10]. The arrival of large groups of refugees into a given country can affect TB control in receiving countries by significantly increasing the disease burden and cost of health services. Moreover, migrant and refugee communities have special health needs and experience obstacles to accessing health care [9].

The number of refugees exceeded 100 million in 2022, which was the highest level that the UNHCR has seen in its almost 75 years [11]. Sub-Saharan Africa hosted more than 26% of the world’s refugee population, and the number of refugees in the eastern African region exceeded 3 million, with the highest in Uganda (1.5 million) [11]. Ethiopia has a long history of hosting refugees and maintains an open-door asylum policy, giving humanitarian access and protection to those seeking refuge [12]. At the end of June 2021, Ethiopia was the third-largest refugee-hosting country in Africa and hosted 785,322 registered refugees, primarily from neighboring countries such as South Sudan, Sudan, Eritrea, and Somalia [11]. Most of the refugees depend largely on humanitarian assistance for their livelihood, and they are accommodated in 26 refugee camps with limited services [13]. In a retrospective study conducted in refugee camps in Ethiopia, TB case notification among refugees, continuously increased from 138 cases in 2014 to 588 cases in 2017 [14]. In another study in the Gambella regional state of Ethiopia, the trend of all forms of TB progressively increased among refugees, contrary to the surrounding communities, in the course of the study period (χ^2^ = 207.7; p < 0.0001) [15]. The current Ethiopian national guidance [16] on programmatic and clinical management of TB could hasten efforts towards the control of TB epidemics in the country. However, it lacks key strategies to control TB in refugee camp settings in Ethiopia. However, except for the above-mentioned studies, there is a shortage of data on the prevalence of TB and associated risk factors among refugees in Ethiopia. Due to the diverse and mobile nature of refugees, the current national TB surveillance mechanisms in Ethiopia often do not capture the prevalence of TB and associated factors among refugees. The evidence of the prevalence of TB and associated factors in refugees is important input for TB prevention and control in refugees and surrounding communities in Ethiopia. Hence, the objective of this study was to estimate the prevalence of bacteriologically confirmed PTB and assess associated factors in presumptive TB refugees residing in refugee camps in Ethiopia.

## Methods

A facility-based cross-sectional study was conducted in selected refugee camps in Ethiopia from February to August 2021. This study was carried out in 12 refugee camps located in five regions in Ethiopia, namely, Tigray region (Mai Ani camp), Afar region (Asaita camp), Gambella region (Pugnido-nuer, Pugnido-agnwak, Kule, and Terkeidi camps), Benishangul-gumuz region (Sherkole, Bambasi, and Tongo camps), and Somali region (Kebribeyah, Sheder, and Melkadida camps). These refugee camps were selected based on population size, one-year smear positivity data [14] from refugee camp clinics, and the representation of refugees from four neighboring countries of Ethiopia (South Sudan, Sudan, Eritrea, and Somalia), where 99% of the refugees in Ethiopia originated [11]. Each refugee camp has refugees from only one country of origin. All presumptive TB cases who attended the selected refugee camp clinics for TB diagnosis during the study period and fulfilled the eligibility criteria were the study population.

The sample size was estimated based on the minimum required sample size for prevalence studies [17]. We used 1.9%, the true population proportion of TB among Somali refugees in Eritrea [7], as a point prevalence estimate of TB. Thus, by considering a one-sided 95% confidence interval; 1.9% (0.02), the true population proportion, and 1% margin of error [17], the required sample size was 530. Assuming 15% non-response due to various reasons (poor documentation, culture contamination, sample rejection, and others), the required total sample size for this study was 610. The total sample size was allocated to the four refugee camp complexes of the country of origin (South Sudan, Sudan, Somalia, and Eritrea) based on their population size in the camp using the proportional allocation method. Thus, 107, 147, 324, and 32 participants were allocated for refugees originating from Eritrea, Somalia, South Sudan, and Sudan, respectively. Sociodemographic data, behavioral characteristics, environmental conditions, and clinical data on TB symptoms were collected consecutively from presumptive TB refugees who attended TB clinics for TB diagnosis.

Refugees originating from Eritrea, Somalia, South Sudan, and Sudan who resided in the refugee camps in Ethiopia; refugees who either had TB-like signs and symptoms or were in contact previously with TB patients; and refugees who were referred to refugee clinics for TB diagnosis were included in the study, whereas presumptive extrapulmonary TB cases and participants younger than 12 years of age were not included in the study due to difficulty coughing and breathing during the sputum induction process.

### Data collection procedure

At each of the refugee camp clinics, arrangements were made with site study coordinators and laboratory professionals. Data collection training and the necessary logistics to collect data were provided at EPHI before the enrollment of study participants. Before the collection of specimens, eligible study participants were provided with all the necessary information. First, written informed consent (from study participants older than 18 years of age) or assent (from study participants aged from 12 to 18 years) was obtained. Then, the study participants were interviewed using the pretested questionnaire designed to obtain socio-demographic data, behavioral characteristics, environmental conditions, and clinical data.

### Specimen collection, storage, and transportation

Two sputum samples (a 5 ml spot and a 5 ml morning) were collected from each study participant in a sterile screwcap universal disposable container provided for this purpose. The study participants were informed to be in a ventilated area dedicated to sputum sample collection. The sputum samples were kept in a refrigerator at a maximum temperature of + 4^o^C until transported to the NTRL using the national postal service system. The sputum samples were placed in a package consisting of a leak-proof primary receptacle (a 50 ml falcon tube), leak-proof secondary package (the container that holds falcon tubes), and rigid outer package as per WHO category B packaging standard [18]. The packages are constructed and closed to prevent any loss of contents that might be caused under normal transportation conditions by vibration or changes in temperature, humidity, or pressure [18]. The specimens were delivered to EPHI within 4 days of collection at the maximum.

### Laboratory investigation and quality control

Direct Xpert MTB/RIF testing was done from the spot sputum sample. The sample preparation for spot sputum was done with sample diluents for 15 min, and 2 ml of the specimen was transferred into the Xpert MTB/RIF cartridge (Cepheid, batch number: 28003). and entered into the GeneXpert instrument (Cepheid, serial number: 808818). By starting the test on the system software, GeneXpert automates all the following steps, including sample work-up, nucleic acid amplification, detection of the target sequence, and result interpretation, within two hours. The Xpert MTB/RIF includes two internal quality controls (sample processing control and probe check control) that verify specimen processing, the success of the polymerase chain reaction, and cartridge integrity [19]. Morning sputum samples were processed for digestion, decontamination, and concentration. Smear preparation was performed from the sediment with the sterile loop. Staining and microscopy were performed for acid-fast bacilli (AFB) using the Zeihl-Nelson (ZN) method. Then the sediment was neutralized and resuspended in 1 ml phosphate buffer solution and inoculated to both LJ media (Becton Dickinson (BD), batch number: 0084401) and MGIT media (Middlebrook 7H9 broth base, BD, batch number: 1,230,007). The remaining sediment was stored in the refrigerator at -80 °C. Inoculated LJ media was incubated at 35–37 °C for 6–8 weeks, with weekly examination for growth. The inoculated liquid media was loaded into the MGIT 960 machine (BD, serial number: MG4193) for incubation and growth of the culture. The machine can detect fluorescence in a liquid culture medium enriched with oxygen to indicate the presence of bacteria. Sterile distilled water was processed for quality control as a start and end control, and results are expected to be negative [20, 21].

To differentiate whether the growth is due to contamination with other microorganisms or true MTB growth, inoculation on blood agar and AFB staining using the ZN method were performed. Finally, the rapid TB antigen (SD Bioline) was done to confirm whether the infection is *Mycobacterium tuberculosis complex* or NTM. The known positive and control strain, H37rv ATCC ®27,294 was tested for rapid TB antigen per new lot [21, 22]. The confirmed MTB isolates were stored in a cryovial tube in the refrigerator at -80 °C.

### Operational definition

New case: refers to a patient who has never been treated for TB or has taken anti-TB drugs for less than 1 month [23]. Previously treated case: refers to a patient who previously received treatment for TB more than a month with treatment outcome of cured and treatment completed, treatment failed, lost to follow-up or not evaluated [23].

### Data processing and statistical analysis

Data were coded, and double data entry was done using EpiData version 3.1. Computerized data were exported to STATA software version 14 for statistical data analysis. Descriptive statistics were used to summarize the study variables. The prevalence of bacteriologically confirmed TB was expressed as a percentage. Factors associated with TB positivity among refugees were investigated using the logistic regression model. The odds ratio and 95% CI around these estimates were used as a measure of the strength of the association. Variables included in the multivariable model were selected via a stepwise procedure based on the likelihood ratios; non-significant independent variables (p-value greater than 0.05) were removed from the logistic regression model. Assumptions such as multicollinearity using the variance inflation factor (VIF) and goodness of fit test such as the Hosmer-Lemeshow test using chi-square were assessed for regression model fitness.

## Results

### Socio-demographic characteristics

A total of 610 eligible refugees were participated in this study. The countries of origin of the study participants were South Sudan (324, 53.1%), Somalia (147, 24.1%), Eritrea (107, 17.5%), and Sudan (32, 5.3%). More than half of the participants were female (54.9%), the mean age was 37.9 years (SD, 16.64), with age range of 12 to 98 years, and 53.6% had no formal education. The larger proportion of the study participants were housewives (37.6%), students (22.8%), and farmers (22.6%) (Table 1).

**Table 1:** Socio-demographic characteristics of 610 presumptive TB refugees residing refugee camps of Ethiopia

Socio-demographic Characteristics	Category	Frequency	Percentage
Country of origin	Eritrea	107	17.5
	Somalia	147	24.1
	South Sudan	324	53.1
	Sudan	32	5.3
Age	12–18	69	11.3
	19–38	265	43.4
	39–58	180	29.5
	> 58	96	15.7
Sex	Male	275	45.1
	Female	335	54.9
Marriage	Single	139	22.8
	Married	388	63.6
	Divorced	19	3.1
	Widowed	64	10.5
Level of Education	No Formal Education	326	53.4
	Grade 1–8	138	22.6
	Grade 9–12	135	22.1
	College and above	11	1.9
Previous Occupation	Farmer	123	22.2
	House wife	208	37.6
	Student	126	22.8
	Teacher	8	1.5
	Health care worker	4	0.7
	Police/Military	10	1.8
	Merchant	13	2.4
	Self-employee	61	11.0

### Prevalence of bacteriologically confirmed TB

**Fig. 1:**
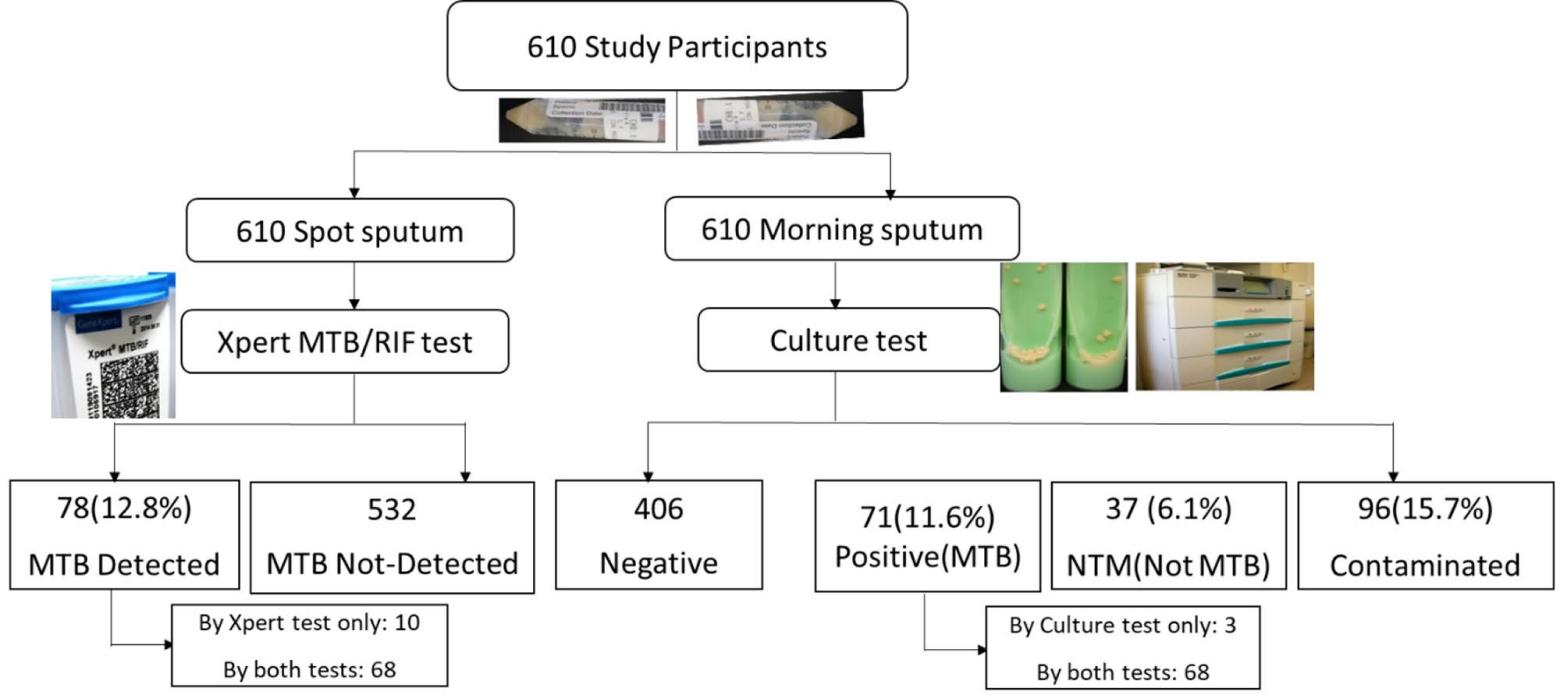
Flow chart of sputum samples and laboratory results (Fig. 1). The percent agreement between Xpert MTB/RIF and culture tests was 97.3% (95CI, 95.4–98.5%).

Of 610 spot sputum samples, MTB was detected in 78 (12.8%, 95% CI, 10.2–15.7%) by Xpert MTB/RIF. Of the 610-morning sputum samples, 71 (11.6%, 95% CI, 9.2–14.5%) were culture positive, 37 (6.1%) were NTM, 96 (15.7%) were contaminated, and the remaining 406 (66.6%) were culture negative. ZN smear microscopy was done from decontaminated sputum sediments, and 39 (6.4%) were smear-positive. The overall prevalence of bacteriologically confirmed PTB among presumptive TB study participants was 13.3% (95% CI, 10.7–16.2) (Fig. 1 ).

The country of origin of the refugees was significantly associated (P-value: 0.005) with bacteriologically confirmed TB cases. The highest proportion of MTB-positive cases were identified from South Sudan, 57 (17.6%), and Somalia, 16 (10.9%) refugees. More males than females were affected by TB (19% vs. 8.7%, p-value: <0.001), and more TB cases were identified in the 19–38 years age group (20%, p-value: <0.001) (Table 2).

**Table 2:** Association of independent variables with bacteriological confirmed TB cases (n = 81)

*Bacteriologically confirmed TB*					
Independent Variables	Category	Total	MTB n (%)	Chi-square	P-value
Country of Origin	Eritrea	107	6(5.6)	12.80	0.005
	Somalia	147	16(10.9)		
	South Sudan	324	57(17.6)		
	Sudan	32	2(6.3)		
Age category	12–18	69	4(5.8)	18.84	< 0.001
	19–38	265	53(20.0)		
	39–58	180	15(8.3)		
	> 58	96	9(13.0)		
Sex	Male	275	52(18.9)	13.79	< 0.001
	Female	335	29(8.7)		
Treatment classification	New case	549	67(12.2)	5.51	0.019
	Previously treated case	61	14(23.0)		
HIV status	Negative	302	60(19.9)	23.82	< 0.001
	Positive	63	7(11.1)		
	Unknown	245	14(5.7)		
BMI	Underweight	352	58(16.5)	7.98	0.019
	Normal	233	22(9.5)		
	Overweight	25	1(4.0)		
Current cigarette smoker	Smoker	139	25(18)	3.46	0.063
	Non-smoker	471	56(11.9)		
Overcrowding (living in < 9m2 tent with households)	Overcrowded	584	79(13.5)		
	Not overcrowded	26	2(7.7)	0.74	0.391
Availability of openable window	Window available	444	63(14.2)	1.18	0.278
	No window	166	18((10.8)		
No of household	1–5	306	42(13.7)	3.19	0.203
	6–10	271	38(14)		
	> 10	33	1(3)		

BMI: body mass index; HIV: Human Immunodeficiency Virus

### Summary of findings from logistic regression

After adjusting for potential confounding variables in the multivariable logistic regression model, bacteriologically confirmed TB remained statistically associated with South Sudan origins (adjusted odds ratio, AOR = 7.74; 95% CI, 3.05–19.64), age group, 19–38 years old (AOR = 5.66; 95% CI, 1.86–17.28), and male sex (AOR = 2.69; 95% CI, 1.58–4.57) participants (Table 3). Multicollinearity and heteroskedasticity were assessed for model fitness. Assumptions were assessed for the logistic regression model fitness. No multicollinearity was detected, and the Hosmer-Lemeshow test revealed the fitness of the model. Hence, the logistic regression model was fitted for the investigation of the independent variables associated with TB positivity.

**Table 3:** Associations of characteristics of study participants with TB occurrence (MTB, n = 81)

Variables	Total	MTB, n (%)	Not-MTB, n (%)	COR (95% CI)	P-value	AOR (95% CI)	P-value
**Country of Origin**					0.008		< 0.001
Eritrea	107	6(5.6)	101 (94.4)	1.00		1.00	
Somalia	147	16(10.9)	131 (89.9)	2.06 (0.78–5.44)		1.95(0.71–5.35)	
South Sudan	324	57(17.6)	267 (82.4)	3.59 (1.50–8.59)		7.74(3.05–19.64)	
Sudan	32	2(6.3)	30 (93.7)	1.12 (0.22–5.85)		1.48(0.27–8.12)	
**Age category**					0.001		0.001
12–18	69	4(5.8)	65 (94.2)	1.00		1.00	
19–38	265	53(20.0)	212 (80.0)	4.06 (1.42–11.65)		5.66(1.86–17.28)	
39–58	180	15(8.3)	165 (91.7)	1.48 (0.47–4.62)		2.12(0.64–7.02)	
>58	96	9(13.0)	87 (87.0)	1.68(0.50–5.70)		2.41(0.67–8.70)	
**Sex**					< 0.001		< 0.001
Male	275	52(18.9)	223 (81.1)	2.46 (1.51-4.00)		2.69 (1.58–4.56)	
Female	335	29(8.7)	306 (91.3)	1.00		1.00	
**Treatment classification**					0.021		0.058
New-case	549	67(12.2)	482 (87.8)	1.00		1.00	
Previously treated case	61	14(23.0)	47 (77.0)	2.14 (1.12–4.10)		2.02 (0.98–4.19)	
**HIV status**					< 0.001		< 0.001
Negative	302	60(19.9)	242 (80.1)	1.00		1.00	
Positive	63	7(11.1)	56 (88.9)	0.50 (0.22–1.16)		0.21 (0.08–0.52)	
Unknown	245	14(5.7)	231(94.3)	0.24 (0.13–0.45		0.20 (0.10–0.39)	
**BMI**					0.023		0.715
Underweight	352	58(16.5)	294 (83.5)	4.74 (0.63–35.70)		2.31 (0.27–19.45)	
Normal	233	22(9.5)	211 (90.5)	2.50 (0.32–19.40)		2.04 (0.24–17.15)	
Overweight	25	1(4)	24 (96.0)	1.00		1.00	
**Current cigarette- smoker**					0.065		0.231
Smoker	139	25(18)	114 (82.0)	1.63 (0.97–2.72)		1.44 (0.79–2.61)	
Non-smoker	471	56(11.9)	415 (88.1)	1.00		1.00	
**Overcrowding (living in < 9m2 tent with households)**					0.398		0.601
Overcrowded	584	79(13.5)	505 (86.5)	1.88 (0.44–8.10)		1.55 (0.30–8.06)	
Not-overcrowded	26	2(7.7)	24 (92.3)	1.00		1.00	
**Availability of openable Window**					0.280		0.185
Window available	444	63(14.2)	381 (85.8)	1.36 (0.78–2.37)		1.56(0.81–3.01)	
No window	166	18((10.8)	148 (89.2)	1.00		1.00	
**No of household**					0.275		0.395
1–5	306	42(13.7)	264 (86.3	5.09 (0.68–38.26)		4.17(0.52–3.61)	
6–10	271	38(14)	233 (86)	5.22 (0.69–39.33)		4.21(0.53–3.48)	
>10	33	1(3)	32 (97)	1.00		1.00	

AOR: adjusted odds ratio; BMI: body mass index; COR: crudes odds ratio; CI: confidence interval; HIV: Human Immunodeficiency Virus

## Discussion

This study aimed to estimate the prevalence of PTB and investigate factors associated with TB positivity among refugees residing in refugee camps in Ethiopia. The current findings revealed that the prevalence of bacteriologically confirmed PTB among presumptive TB refugees was 13.3%. The country of origin of the refugees was significantly associated with bacteriologically confirmed TB cases. The highest proportion of MTB-positive cases were identified from South Sudan, 57 (17.6%), and Somalia, 16 (10.9%) refugees. The multivariable logistic regression model showed that bacteriologically confirmed TB remained statistically associated with South Sudan origins, younger age group (19-38 years old) and male sex participants.

The current study reported a high prevalence of PTB among presumptive TB cases. Similar studies [14, 15] reported a higher magnitude and increasing trends of TB among refugees in Ethiopia. In a study [14] done in refugee camps in Ethiopia, TB case notification continuously increased from 138 cases to 588 cases (from 2014 to 2017), and in another study [15] in the Gambella region of Ethiopia, notified TB cases among refugees showed a 29.0% increase from 2009 to 2017. Both studies were based on retrospective data, which might have compromised the quality. Moreover, the methods of TB diagnosis used were sputum smear microscopy and clinical diagnosis, which might decrease the proportion of TB cases.

Our study findings were comparable with facility-based TB prevalence studies in Ethiopia [24–27]. The prevalence of PTB among presumptive TB patients presenting cough > 2 weeks in Addis Ababa was 11.9% [24] using the culture-based method. This finding [24] is similar to our study results (11.6%) using a culture-based method. A similar prevalence (13.5%) was observed in the study done at St. Peter Specialized Hospital, Addis Ababa, Ethiopia [25]. However, a relatively higher prevalence (15.1% vs. 13.3%) was observed in the study [26] done in presumptive TB cases in the government hospital in Addis Ababa, Ethiopia. Similarly, a relatively higher prevalence (16.7% vs. 13.3%) was observed in a multicentral study [27] in Ethiopia. These might be due to the data and sample collection methods used, which might increase the prevalence of PTB. However, a slightly higher prevalence (13.3% vs. 12%) was revealed in our study compared to the Ethiopian national annual TB performance report [28]. This is because our study used both culture and Xpert MTB/RIF, which might increase the total TB cases detected during the study period.

A relatively higher prevalence (13.3% vs. 10.7%) was reported in the current study compared to the prevalence of TB among Syrian refugees sheltered in Turkey [29]. The Syrian war has forced millions of Syrians to seek refuge in neighboring countries, thus contributing to an increase in the number of TB cases across the region [29]. Similarly, higher TB cases were detected in 6 months in this study compared to the TB cases that were detected in Syrian refugees in Jordan in 16 months [29, 30]. This could be due to the lower TB prevalence reported by Syria (23/100,000), Jordan (6/100,000), and Turkey (22/100,000) before the onset of the Syrian crisis. The proportion of TB among Syrian refugees in Lebanon [29] was higher compared to the current study. This could be explained partly by the absence of formal refugee camps in Lebanon, where refugees were sheltered in the communities across the nation. This could increase the transmission of TB, which would further increase the proportion of TB in refugees.

The highest prevalence (535/100 000) was reported from asylum seekers in Italy [31] compared to our study. The prevalence was found to be 80 times greater compared to the estimated prevalence rate in Italy (6.7 per 100, 000 population). While in Europe, the majority of reports indicated low prevalence (20/100,000 population) [32]. This may be explained by extended stays in detention and overcrowding for several months on migration routes before reaching the Italian coasts. These might increase the risk of TB transmission and the progression from infection to disease. In contrast, the screening of migrants by adopting a chest radiograph with a specific TB questionnaire might overestimate the TB screening result [31].

The proportion of TB cases in our study is comparable with the study conducted in Malta [33]. TB notification rates ranged from 470 to 880 per 100, 000 population among migrant boat arrivals from 2007 to 2011. Bacteriological evidence was used for TB notification, and 89 TB cases were notified, which is similar to our study (81 TB cases). In another study [34] higher notification of TB cases was detected (17.4%) compared to our result (13.3%). The higher notification of TB cases might be due to the data collection from routine case-based surveillance, particularly overreporting as a result of a lack of representativeness and lack of timeliness. A low (0.2%, 11 TB cases) TB notification rate was reported in a study [35] done in Italy compared to our study. This could be due to the diagnostic evaluation used, which might be a highly specific method that might reduce the TB notification rate.

The country of origin and the local epidemiology are important factors when considering TB prevalence in migrants and refugees [36]. In 2020, the TB incidences in the countries of origin were Eritrea (81/10^5^), Somalia (259/105), South Sudan (232/10^5^), and Sudan (63/10^5^) [37]. The above origin of country incidence profile was consistent with our findings, in which high MTB-positive cases were identified from South Sudan (17.6%) and Somalia (10.9%) refugees in our study. Thus, our findings represent the magnitude of TB in the country of origin. The higher number of bacteriologically confirmed TB cases in our finding might be due to the fact that most of the refugees originate from countries with a high TB burden [7]. This suggests that TB screening strategies in cross-border areas and in refugee camps might be a good approach to tracing refugees from high TB-burden countries. Furthermore, the national TB program needs to strengthen TB prevention and control activities in the refugee population and surrounding communities in Ethiopia.

TB affects all age groups [37], but disproportionally affects certain age groups. More TB patients were observed in younger age groups < 35 years in a study [38] and 15–44 years in another study [14]. Similarly, more TB cases were identified in the younger age group, 19–38 years, in the current study. TB affects people of both sexes, but the highest burden is in adult men [14, 37, 38]. One explanation could be that men have greater risk behavior than women [39]. Although 55% of the participants in our study were women refugees, males were more affected by TB than females (19% vs. 8.7%). A multivariable analysis of this study showed that male sex was considered an associated risk factor for TB among refugees in refugee camps in Ethiopia. This finding is consistent with other studies, which have shown that the risk of TB remains increased with, male sex [40]. In the studies, the age group [26, 29] and country of origin [40, 41] showed significantly associated factors for TB. Similarly, the multivariable analysis of this study also revealed South Sudan origins and a younger age group (19–38 years old) as significantly associated risk factors for bacteriologically confirmed TB among refugees in refugee camps in Ethiopia. Our findings from the multivariate analysis suggest that more attention should be given to South Sudan origins, male sex, and younger age group (19–38 years old) in the screening and detection of TB in the refugee camps in Ethiopia. Human Immunodeficiency Virus (HIV) is the well-known risk factor for developing active TB disease [1, 42]; however, no significant association was found in the current study. This could be due to the high number (245/610) of participants with unknown HIV-status, which might decrease the number of HIV-positives in the current study. The HIV-status of the participants was known from interviews during the data collection period of the current study. Overcrowding and malnutrition, which are risk factors for TB in refugees [7, 43], were not significantly associated with TB in our study. This might be due to the limited number of confirmed TB cases (81) and the other reason could be that our study determined overcrowding in households living in < 9m^2^ tent and malnutrition (underweight) in BMI < 18.5 kg/m^2^. This might need further investigation. Furthermore, additional studies should be recommended to study the associated factors of TB in the refugee population.

The strengths of this study were the representation of all four countries of origin of refugees, which represented 99% of refugees in Ethiopia; and the laboratory investigation by both culture-based and molecular methods. The limitation of this study was the passive prevalence study method used, in which the presumptive refugees enrolled in the study after they were referred to refugee clinics for diagnosis.

## Conclusion

The prevalence of bacteriologically confirmed PTB among presumptive TB refugees residing in refugee camps in Ethiopia was high. South Sudan origins, younger age group (19–38 years old) and males were found to be significantly associated factors for bacteriologically confirmed TB among refugees residing in refugee camps in Ethiopia. The findings of this study could benefit the national TB program, which can aid TB prevention and control activities in the refugee camps and surrounding communities in Ethiopia. We recommend that TB prevention and control activities should be strengthened and an active TB survey program should be implemented in refugee camps, surrounding communities, and border crossing areas in Ethiopia.

## Data Availability

All data and materials that support the final results are presented in the manuscript.

## List of abbreviations

AAU: Addis Ababa University
AFB: acid fast bacilli
ALIPB: Aklilu Lemma Institute of Pathobiology
ARRA: Agency for Refugees and Returnees Affairs
BD: Becton Dickinson
EPHI: Ethiopian Public Health Institute
HIV: Human Immunodeficiency Virus
LJ: Lowsteen Jensen
*M. tuberculosis*: *Mycobacterium tuberculosis*
MGIT: Mycobacterium Growth Indicator Tube
MTB: *Mycobacterium tuberculosis*
NTM: Non-tuberculous mycobacteria
NTRL: National Tuberculosis Reference Laboratory
PTB: pulmonary TB
RIF: Rifampicin
TB: Tuberculosis
UNHCR: United Nations High Commission for Refugees
VIF: Variance Inflation Factor
WHO: World Health Organization
ZN: Zeihl-Nelson
