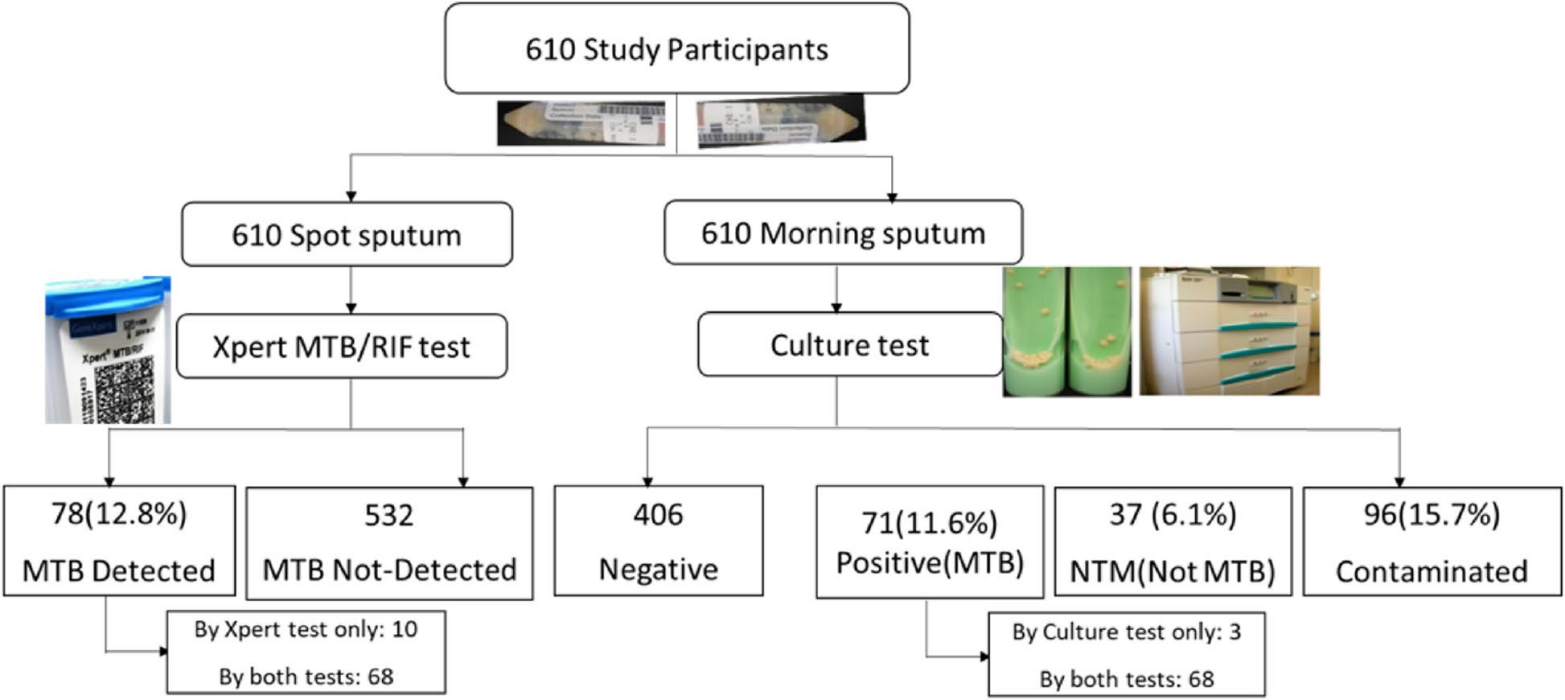# Correction: Prevalence of tuberculosis and associated factors among presumptive TB refugees residing in refugee camps in Ethiopia

**DOI:** 10.1186/s12879-023-08905-6

**Published:** 2023-12-20

**Authors:** Abyot Meaza, Bazezew Yenew, Miskir Amare, Ayinalem Alemu, Michael Hailu, Dinka Fikadu Gamtesa, Mirgissa Kaba, Girmay Medhin, Gobena Ameni, Balako Gumi

**Affiliations:** 1 Aklilu Lemma Institute of Pathobiology (ALIPB), Addis Ababa University (AAU), P.O. Box 1176, Addis Ababa, Ethiopia; 2 Ethiopian Public Health Institute (EPHI), Swaziland Street, PO Box 1242, Addis Ababa, Ethiopia; 3 School of Public Health, Addis Ababa University, Addis Ababa, Ethiopia; 4 Department of Veterinary Medicine, College of Agriculture and Veterinary Medicine, United Arab Emirates University, PO Box 15551, Al Ain, UAE

**Correction: BMC Infect Dis 23, 498 (2023)**

**https://doi.org/10.1186/s12879-023-08469-5**

The original publication of this article [1] contained 2 errors in Fig. 1:

- The box describing "532 MTB Detected" should be "532 MTB Not-Detected"
- "Morning" should be 610 Morning sputum"

The incorrect and correct figure are shown in this correction article. The original article has been updated.

**Incorrect Figure 1**


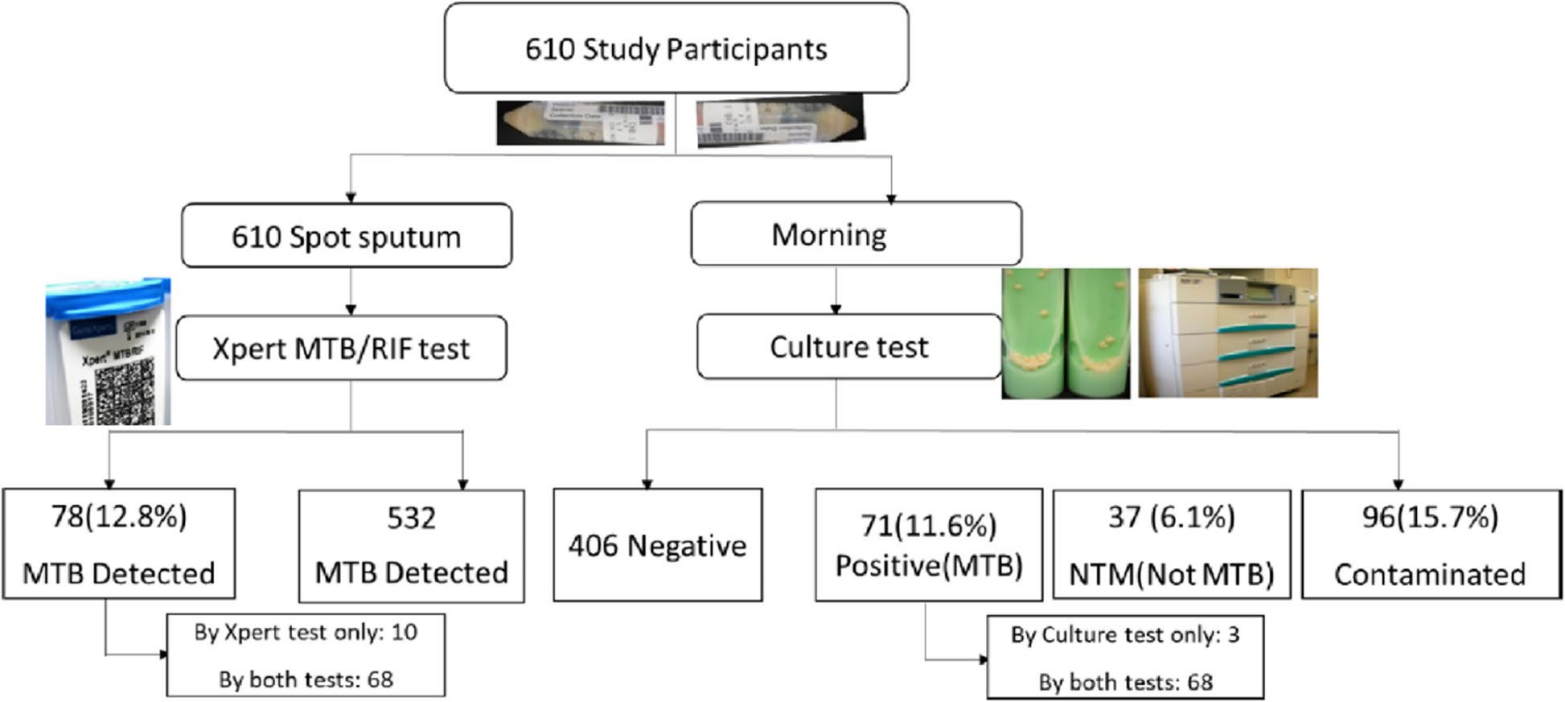


**Correct Figure 1**